# Implementation of a new guideline in cardiovascular secondary preventive care: subanalysis of a randomized controlled trial

**DOI:** 10.1186/s12872-016-0252-0

**Published:** 2016-04-30

**Authors:** Stina Jakobsson, Daniel Huber, Fredrik Björklund, Thomas Mooe

**Affiliations:** Department of Public Health and Clinical Medicine, Östersund, Umeå University, Umeå, Sweden; Bergsundsgatan 23, 11737 Stockholm, Sweden

**Keywords:** Acute coronary syndrome, Cardiovascular disease, Myocardial infarction, Randomized controlled trial, Secondary prevention, Stroke, Transient ischemic attack

## Abstract

**Background:**

Cardiovascular secondary preventive recommendations are often not reached. We investigated whether a nurse-led telephone-based follow-up could improve the implementation of a new guideline within a year after its release.

**Methods:**

In February 2013, a new secondary preventive guideline for diabetic patients was released in the county of Jämtland, Sweden. It included a changed of the low-density lipoprotein cholesterol (LDL-C) target value from <2.5 mmol/L to <1.8 mmol/L. In the Nurse-Based Age-Independent Intervention to Limit Evolution of Disease (NAILED) trial, patients with an acute coronary syndrome, stroke, or transient ischemic attack were randomized to secondary preventive care with nurse-based telephone follow-up (intervention) or usual care (control). Patient data were obtained from the NAILED trial to study the implementation of the new LDL-C guideline by comparing telephone follow-up with usual care. The Mann–Whitney *U*-test was used for continuous variables, and Person’s *χ*^2^ test was used for categorical variables to assess between-group differences.

**Results:**

Out of the 1267 patients that entered the study period, 101 intervention and 100 control patients with diabetes fulfilled the inclusion criteria and completed the study period. Before the guideline change, 96 % of the intervention patients and 70 % of the control patients reached the target LDL-C value (*p* < 0.001). After the guideline change, the corresponding respective proportions were 65 % and 36 % (*p* < 0.001). The main reason that intervention patients did not achieve the target LDL-C value was that they received full-dose treatment; for control patients, the main reason was that medication was not adjusted, for an unknown reason.

**Conclusions:**

One year after a change in the cardiovascular secondary preventive guideline, nurse-based telephone follow-up performed better than usual care to implement the new recommendation.

**Trial registration:**

ISRCTN registry; ISRCTN96595458 (date of registration 10 July 2011) and ISRCTN23868518 (date of registration 13 May 2012).

## Background

Treatment of modifiable risk factors after a cardiovascular event is of great importance. The combination of different therapies can reduce the risk of subsequent events substantially [1]. Consequently, treatment guidelines for secondary prevention in patients with established cardiovascular disease (CVD) have been published, addressing the CVD population in general, as well as different subsets of patients with specific comorbidities. As evidence emerges to support new treatment strategies, international, national, and local updates of these guidelines are frequently released [2–5]. In general, physicians seem to have positive attitudes towards guidelines, and the self-reported adherence is high [6–8]. However, previous investigations of CVD patients’ modifiable risk factors have found that treatment goals according to guidelines are often not reached; consequently, optimum risk reduction is not achieved [9–12]. These previous studies investigated adherence to guidelines at a substantial interval after they had been released, and less is known about adherence during the first year after guideline release. Knowledge about guideline implementation is important, because rapid adjustment provides an opportunity to reduce the risk of recurrent events.

In Sweden, the general practitioner (GP) usually manages secondary preventive follow-up after hospital care for a cardiovascular event. Local guidelines for the treatment of diabetic patients in primary health care in the county of Jämtland, Sweden, were changed in February 2013. A GP was responsible for the revision of the guidelines, and a new recommendation was included regarding low-density lipoprotein cholesterol (LDL-C) for diabetic patients with established CVD. To better agree with the European guidelines [3], the target level was lowered from LDL-C <2.5 mmol/L to <1.8 mmol/L.

The Nurse-Based Age-Independent Intervention to Limit Evolution of Disease after Acute Coronary Syndrome (NAILED ACS) and the NAILED stroke risk factor trials are ongoing randomized controlled trials in the county of Jämtland. Their objective is to improve secondary prevention after acute coronary syndrome (ACS), stroke, and transient ischemic attack (TIA) through a nurse-based telephone follow-up concerning cardiovascular risk factors. Different organizational strategies have previously been evaluated to improve secondary preventive care (e.g., patient education, physician education, pre-booked doctor appointments, etc.), most of which have not proven effective with respect to risk factor reduction [13, 14]. There are some exceptions to this, including some support for telephone-based follow-up to improve risk factor control [15, 16]. The aim of this report was to describe the implementation of new cardiovascular guidelines in primary care and to compare it with the performance of the follow-up in the NAILED study. We hypothesized that the proportion of patients that reached the target LDL-C value the year after the guideline change would decrease, and that a greater proportion of patients randomized to nurse-based, telephone follow-up would reach the new recommended levels of LDL-C, compared with patients randomized to usual care.

## Methods

The study was approved by the Regional Ethics Committee, Umeå University (reference number Dnr-09-142 M). Patient data were obtained from the ongoing randomized, controlled NAILED trials with the aim to improve patients’ blood pressure and LDL-C values. A detailed description of the rationale and design of the NAILED trails has been published [17, 18]. In brief, all patients living in the county of Jämtland, Sweden who are hospitalized with a diagnosis of myocardial infarction, unstable angina, stroke, or TIA are assessed for inclusion. Östersund Hospital is the only hospital in the county; it has a rural catchment area, with a population of approximately 125,000 inhabitants. Study nurses identified eligible patients with the physical and mental capacity to communicate by telephone. Patients with deafness, aphasia, dementia, or severe (often terminal) diseases were not included. Participants in other ongoing trials were also excluded. All eligible patients were informed about the study, and those who agreed to participate were randomized to intervention or control in a 1:1 ratio.

Patient characteristics, including medical history, were recorded during the initial hospitalization. At 1 month after discharge, baseline measurements of blood lipids and blood pressure (BP) were performed. Corresponding follow-up measurements were performed annually thereafter.

Shortly after the baseline blood lipid and BP measurements, participants in both study groups were contacted by a study nurse by telephone and interviewed about their wellbeing and adherence to medical treatment. In the control group, LDL-C and BP values were forwarded directly to the patient’s GP for assessment, without further action from the study team. For patients randomized to intervention who did not reach the target values for LDL-C and/or BP, medication titration was made. Tests were repeated within approximately 4 weeks, and additional medication titration was performed if necessary until target values were reached or no additional changes were considered reasonable. A study physician made decisions regarding titration and medication.

On 14 February 2013, information about a local guideline change regarding target LDL-C value for patients with diabetes mellitus (DM) and established CVD was distributed to all GPs through a joint e-mail group, which is used for spreading information that concerns primary health care in Jämtland. As stated above, the target value for LDL-C was lowered from LDL-C <2.5 mmol/L to LDL-C <1.8 mmol/L, in accordance with the European Society of Cardiology’s guidelines [3]. The same LDL-C target value was adopted 24 March 2013 by the NAILED trial, in order to follow the same guidelines as primary care. No extra follow-up was performed within the NAILED studies because of the guideline change, and the scheduled follow-up according to the study protocol remained the same. All diabetic patients in the NAILED trial database who received a telephone follow-up call before 14 February 2013 (any of 1 month or 1, 2, or 3 years after the initial hospitalization) and another follow-up call after 31 March 2013 and before 16 June 2014 were included in the analysis. We chose to study the implementation of the new guideline after 31 March 2013 to give all the study nurses a chance to receive the new information. The end date of follow-up was set to 15 June 2014, to make sure that all patients had one follow-up and that medication titrations, if any, had been completed.

Baseline characteristics in the current analysis were those reported at the most recent follow-up within the NAILED-trial before 14 February 2013. Patients were considered to have DM if they received glucose lowering medication or dietary treatment at the follow-up after 31 March 2013. If a patient reached LDL-C <1.8 mmol/L at follow-up, that value was reported as the end-point LDL-C. For patients in the intervention group who did not achieve the target LDL-C value, the value reached after medication titration was reported as the end-point LDL-C value. The patient records were scrutinized for those patients in the control group who did not reach target value at the first follow-up after 31 March 2013, in order to determine whether the GP took notice of the elevated LDL-C value, and if so, whether any medication titration was performed. In Jämtland, the standard of care for diabetic patients includes a yearly visit to their GP. During this visit, local guidelines state that risk factor control and intervention regarding established risk factors for CVD is performed. This includes laboratory measurements of serum levels of glucose and lipids, as well as clinical examination including BP control. If no patient record could be found that mentioned the LDL-C value within 1 month after the NAILED-control, then we used the LDL-C value recorded at the first yearly diabetes control after 31 March 2013. If the GP titrated any medication, the value after titration was used. If no patient record was found regarding LDL-C values and no yearly diabetes control had been performed before the end date for the present study, 15 June 2014 (*n* = 8), the LDL-C value at the NAILED trial follow-up was reported. Atorvastatin 80 mg or rosuvastatin 40 mg, with or without ezetimib 10 mg, was considered full-dose treatment.

### Statistical analysis

The results are presented as median and percentiles for continuous variables and as percentages for categorical variables. The Mann–Whitney *U*-test was used for continuous variables since the data was not normally distributed, and Person’s *χ*^2^ test was used for categorical variables to assess between-group differences. The Wilcoxon signed rank test was used to study the change in LDL-C before and after the guideline change. *P* < 0.05 was considered statistically significant. Statistical analyses were performed using SPSS 20.0 software.

## Results

**Fig. 1:**
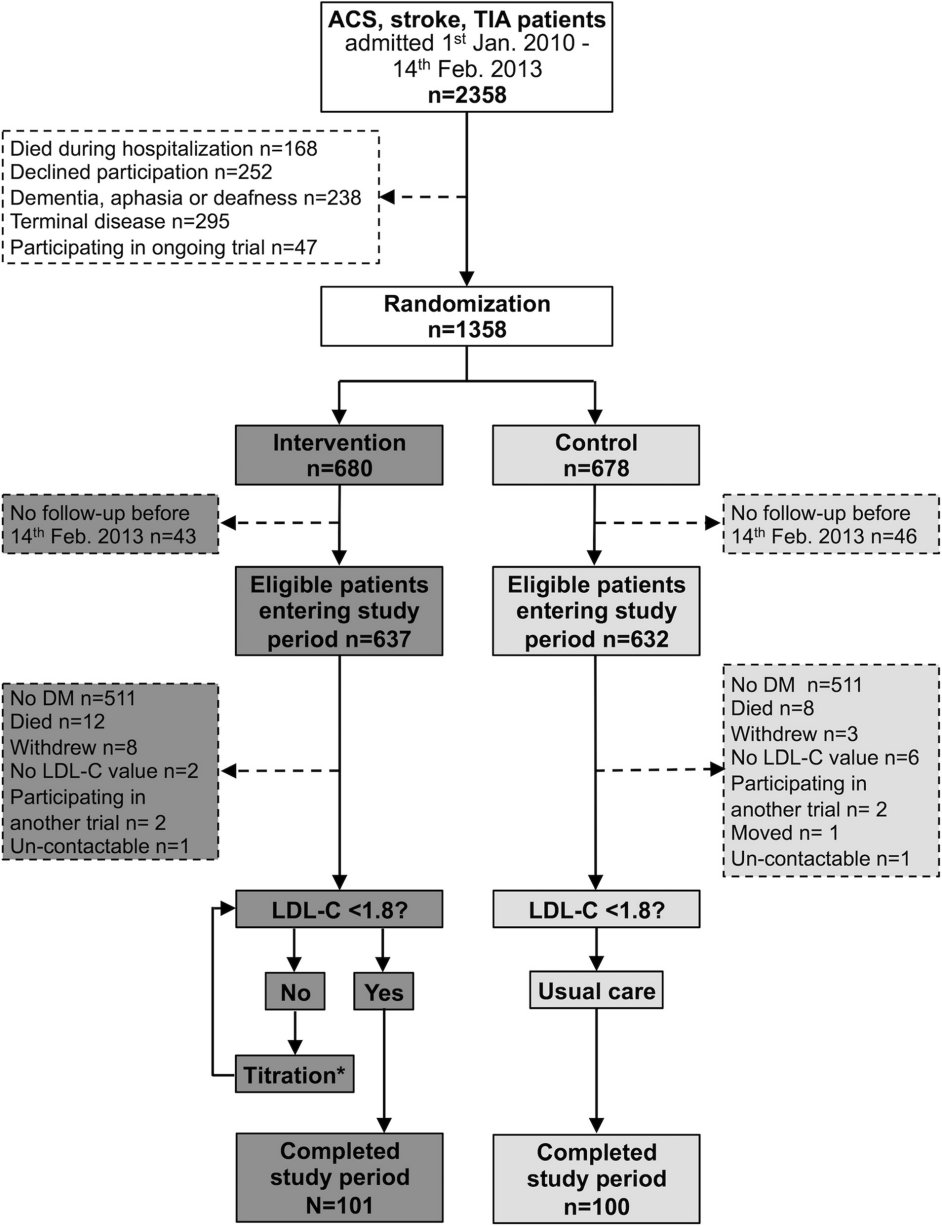
Study flow chart. *If necessary, medication was titrated until target values were reached or until no further changes were considered reasonable. Jan, January; Feb, February; n, number of cases; Mar, March; DM, diabetes mellitus; LDL-C, low-density lipoprotein cholesterol

Two hundred and one ACS, stroke, or TIA patients had at least one follow-up call before 14 February 2013, a follow-up after 31 March 2013 and had diabetes. Of these, 101 patients had been randomized to the intervention and 100 to the control group (Fig. 1). Baseline characteristics are shown in Table 1. The intervention and control groups were well matched except for the cholesterol values and BP (i.e., the variables under intervention in the NAILED study). The intervention group contained significantly more patients with ACS than stroke as the qualifying event than the control group. The randomization process was stratified for ACS and stroke/TIA, but not for diabetes status. The difference in the proportion of ACS patients between the intervention and control groups in the DM cohort was seen already at randomization (75 patients with DM and ACS in the intervention group and 61 in the control group; 53 patients with DM and stroke or TIA in the intervention group and 63 to the control group). The difference between the proportions of ACS and stroke/TIA patients in the DM intervention group was further enhanced by more new cases of diabetes among those with prior ACS (*n* = 12) than among those with a prior stroke or TIA (*n* = 1) who were randomized to intervention. Among those randomized to the control group the number of new diabetes cases was fairly similar among those with a prior stroke or TIA (*n* = 8) or a prior ACS (*n* = 6).

**Table 1:** Baseline characteristics

	Intervention (*n* = 101)	Control (*n* = 100)	*P*-value
Age, years	72 (66–80)	73 (66–79)	0.99
Women (%)	27.7	30.0	0.72
Smoking (%)	4.0	6.0	0.51
Self-reported health^a^	70 (50–85)	70 (50–80)	0.70
Prior cardiovascular disease^b^	38.6	42.0	0.63
HbA1c, mmol/mol	58 (50–70)	57 (51–65)	0.36
Weight, kilograms	85 (75–97)	86 (76–97)	1.0
Waist, centimeters	103 (95–111)	105 (96–112)	0.55
Systolic blood pressure, mmHg	128 (120–133)	134 (124–145)	<0.001
Diastolic blood pressure, mmHg	70 (65–80)	76 (70–82)	0.005
Cholesterol, mmol/L	3.7 (3.4–4.0)	4.1 (3.6–4.6)	<0.001
LDL-C, mmol/L	1.9 (1.5–2.0)	2.1 (1.6–2.5)	<0.001
HDL-C, mmol/L	1.20 (0.98–1.39)	1.18 (0.97–1.48)	0.88
Triglycerides, mmol/L	1.4 (1.0–1.9)	1.5 (1.2–2.2)	0.06
Study, ACS^c^ (%)	64.4	47.0	0.01
No lipid lowering treatment (%)	9.9	12.0	0.63
Simvastatin (%)	47.5	54.0	0.36
Atorvastatin (%)	36.6	32.0	0.49
Other lipid lowering strategy^d^ (%)	5.9	1.0	0.06

If not other specified, values are reported as median (25^th^–75^th^ percentiles)

LDL-C, low density lipoprotein cholesterol; HDL-C, high density lipoprotein cholesterol; ACS, acute coronary syndrome

^a^Self-reported health between 0–100

^b^Previous myocardial infarction, angina pectoris, stroke or peripheral artery disease

^c^Patient included in the study due to ACS or stroke/TIA

^d^Rosuvastatin, combination of rosuvastatin and ezetimib or gemfibrozil

Lipid-lowering treatments after the introduction of the new guideline (before and after titration of medication) are shown in Table 2. In the intervention group, 39.6 % of patients had at least one medication titration. In comparison, 9.0 % of control patients had one or several medication titrations (*p* < 0.001).

**Table 2:** Lipid lowering treatments after the guideline change

	Before medication titration			After medication titration		
	Intervention	Control	*P*-value	Intervention	Control	*P*-value
No treatment (%)	12.9	11.0	0.68	4.0	9.0	0.15
Simvastatin (%)	33.7	52.0	0.009	21.8	51.0	<0.001
Simvastatin 10 mg	2.0	5.0	0.24	1.0	6.0	0.05
Simvastatin 20 mg	14.9	25.0	0.07	9.9	21.0	0.03
Simvastatin 40 mg	16.8	22.0	0.35	10.9	24.0	0.01
Atorvastatin (%)	44.6	35.0	0.17	56.4	38.0	0.009
Atorvastatin 20 mg	6.9	13.0	0.15	5.0	14.0	0.03
Atorvastatin 40 mg	25.7	19.0	0.25	29.7	21.0	0.16
Atorvastatin 60 mg	0	1.0	0.31	0	1.0	0.31
Atorvastatin 80 mg	11.9	2.0	0.006	22.8	2.0	<0.001
Rosuvastatin (%)	4.0	0	0.04	8.9	0	0.002
Rosuvastatin 20 mg	3.0	0	0.08	3.0	0	0.08
Rosuvastatin 40 mg	1.0	0	0.32	5.9	0	0.01
Other^a^ (%)	5.0	2.0	0.25	7.9	2.0	0.05

^a^Pravastatin 40 mg, combination of atorvastatin 80 mg and ezetimib 10 mg or combination of rosuvastatin 40 mg and ezetimib 10 mg

The median LDL-C value before the guideline change from LDL-C <2.5 mmol/L was 1.9 mmol/L in the intervention group and 2.1 mmol/L in the control group (*p* < 0.001). After the introduction of the new target LDL-C value (<1.8 mmol/L) the median values in intervention and control patients were 1.7 mmol/L and 2.0 mmol/L, respectively (*p* < 0.001). The LDL-C value decreased significantly in the intervention group (*p* = 0.03), but not in the control group (*p* = 0.77).

**Fig. 2:**
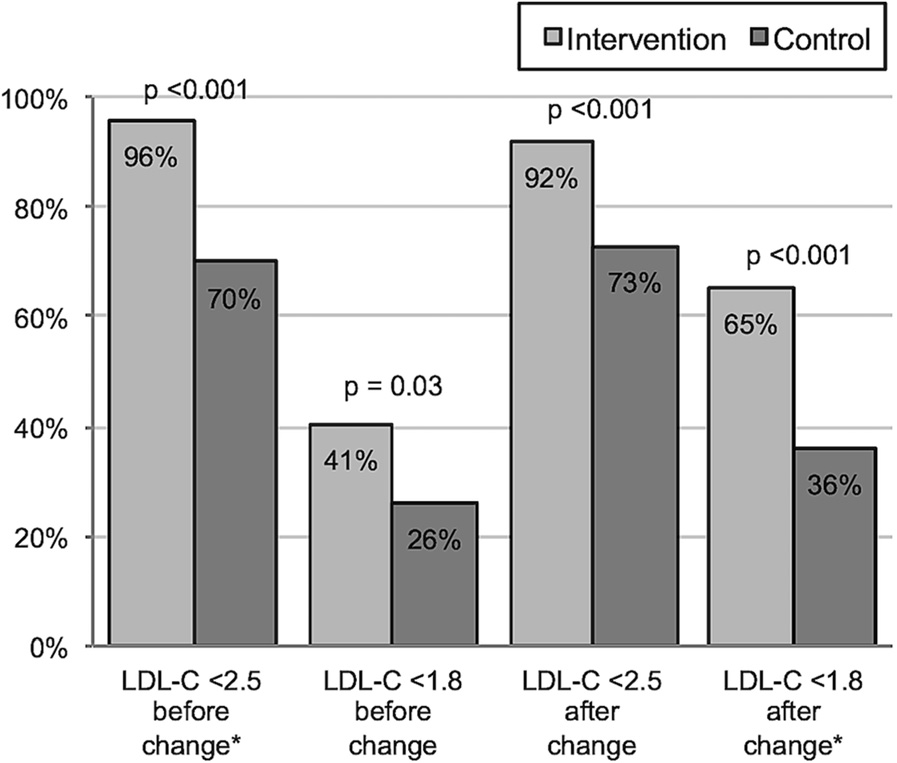
Proportion of patients that achieved the target LDL-C value before and after the guideline change. *The target value at the time being investigated. LDL-C, low-density lipoprotein cholesterol

Figure 2 shows the proportion of patients that reached the target value before and after the guideline change. Before the change, 96.0 % of intervention patients and 70.0 % of control patients reached the target LDL-C value of <2.5 mmol/L (*p* < 0.001). After the change to the new LDL-C target (<1.8 mmol/L), the corresponding proportions of patients were 65.3 and 36.0 %, respectively (*p* < 0.001).

**Fig. 3:**
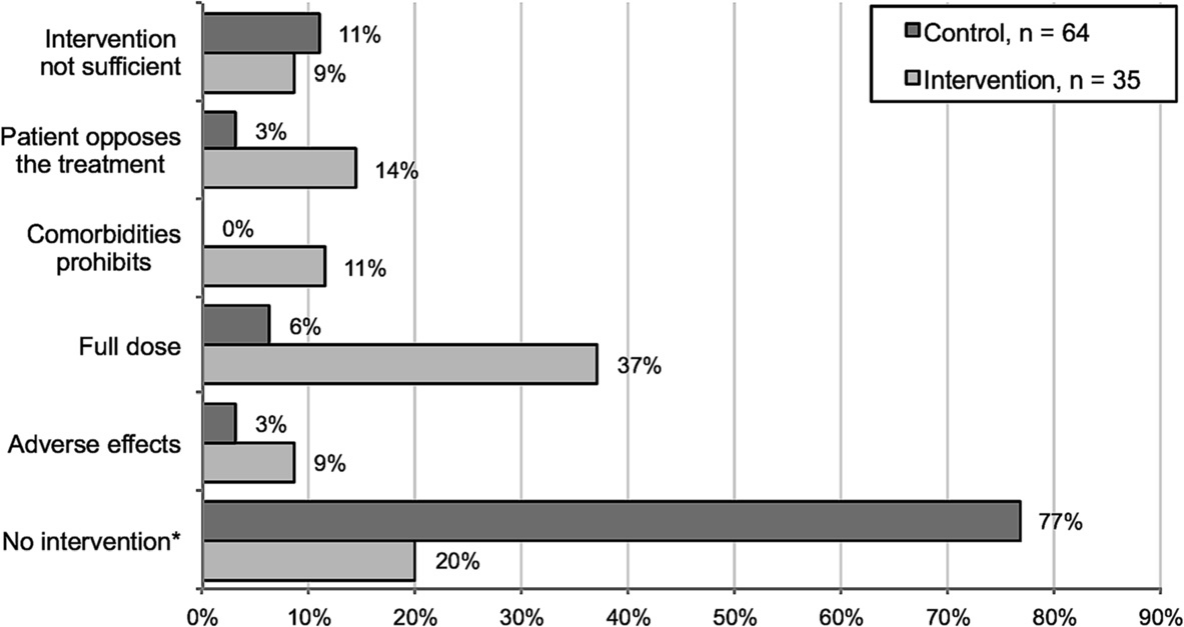
Reported reasons for not reaching the target LDL-C value. *No reason was reported for not performing any intervention

Thirty-five intervention patients and 64 control patients did not reach LDL-C <1.8 mmol/L after the change in the guideline. In the intervention group, the main reasons for failing were that patients were on full-dose treatment (37.1 %) or no intervention was performed for an unknown reason (20.0 %). For control patients, the main reasons for not reaching target value were that no intervention was performed (76.6 %) or that intervention was performed but target value was not reached (10.9 %). All of the reported reasons for not reaching LDL-C <1.8 mmol/L after the guideline change are shown in Fig. 3.

## Discussion

To the authors’ knowledge, this is the first study to assess the early effect on implementation of a new secondary preventive guideline for patients with established CVD and compares a structured, nurse-based intervention with usual care according to current practice. We have shown that, compared with usual care, a secondary preventive, nurse-based telephone follow-up increased the proportion of patients that reached the target level of LDL-C during the first year after a guideline change. The main reason for failing to reach the target value in the intervention group was that the maximal statin dose had been reached. Regarding the vast majority of patients in the usual care group who did not reach the target, no reason was found in their medical records.

Different organizational strategies to improve secondary preventive care for patients with established CVD have been evaluated in previous studies; two recent Cochrane reviews lacked clearly positive results [13, 14]. A key methodological difference between the trials included in the meta-analyses and the method used in the NAILED trial was the prompt titration of medication by a study physician when patients’ LDL-C levels were above target. One meta-analysis that compared different organizational strategies to improve glycemic control in diabetic patients found that the greatest reduction occurred in trials in which case managers were able to adjust medications promptly [19]. McAlister et al. also found that case management after ischemic stroke with active prescribing by a pharmacist improved the control of LDL-C compared with nurse-led screening and feedback to the GP [15].

Trials that evaluated telephone follow-up as a part of secondary preventive care have shown some positive results regarding the control of modifiable risk factors. However, these studies had rigorous protocols, ambitious patient information packages, and frequent patient support, which may have limited their applicability in clinical practice [20–22]. The NAILED trial employed a simplified, nurse-based telephone follow-up protocol. A method for yearly follow-up of modifiable risk factors must be cost-effective, as well as easy to implement throughout healthcare organizations. The simplified NAILED method makes it possible to include a large proportion of eligible patients. For example, living in rural areas is not an obstacle to receiving follow-up by telephone. Furthermore, the median age in the present study was over 70 years, which shows that telephone follow-up according to the NAILED trial can be implemented in an aged patient population with considerable comorbidity. In comparison, the mean age of participants in the previously mentioned telephone follow-up trials was between 59 and 65 years [20–22].

Even though the nurse-based telephone follow-up performed better than usual care, there were still fewer patients in both intervention and control groups who reached the target LDL-C value after the guideline change. In the intervention group, the main reason for not reaching the target LDL-C value was that patients were considered to have full dose treatment. This finding emphasizes that even if patients received atorvastatin 80 mg or rosuvastatin 40 mg, as well as dietary and exercise advice, some patients cannot reach desirable LDL-C values with the treatment options available today. Recently reported results about the effect of adding ezetimibe or PCSK9 inhibitors to statin treatment indicate that the proportion of patients in whom an LDL-C level <1.8 mmol/L can be reached will increase [23, 24].

The intervention patients’ test results were screened by a trained study nurse whose main focus was to ensure that the patients’ risk factors were at a desirable level, and was otherwise supposed to notify a study physician. Despite this, 7/35 (20.0 %) intervention patients who did not reach the LDL-C target received no intervention for an unknown reason. Hence, even under controlled conditions, optimal treatment of risk factors is difficult to achieve, largely because of human factors.

For patients in the control group, the main reason for not reaching the LDL-C target was that the treating physician did not actively intervene to improve LDL-C levels, for an unknown reason. Possible causes for the physicians’ lack of action include lack of time, lack of patient adherence, lack of guideline knowledge, and differing opinions about target value compared with guidelines. These have all been suggested as reasons for not adhering to guidelines in previous studies [8, 25]. Other possibilities could be that the treating physician had forgotten about the new guideline or had not read the information about the guideline change, both of which could be a result of the abundance of guidelines directed to GPs [26].

There were very few cases of adverse effects being reported as the cause for not reaching the target LDL-C value. In the intervention group, this can probably be explained by flexible medication; using different doses and different statins made it possible to overcome many of the perceived adverse effects [27, 28]. In the control group, one possible reason is that patients have previously reported adverse effects caused by lipid-lowering treatments; however, this reason was not found in the medical records reviewed. This would be reported as “No intervention”.

The intervention group contained significantly more patients with ACS than stroke as the qualifying event than the control group. This difference was present already at randomization and was then further increased by more new cases of diabetes in the intervention group among those with prior ACS and in the control group among those with a prior stroke or TIA. Consequently, the unequal distribution of patients with ACS between the intervention and control groups seems to be caused by the play of chance.

Our results lead us to believe that a number of components are needed to achieve adequate implementation of (and optimal adherence to) recommended secondary preventive guidelines; a structured method, comparable with the NAILED method; a procedure for rapid medication titration to reach target values to the greatest extent possible; a system with a control mechanism to ensure that no patients are missed and that the treatment is intensified as needed; and finally, different pharmacological options, in order to overcome potential adverse effects.

### Limitations

This study has some limitations. First of all, the patients who chose to participate in the study might have been more motivated to follow secondary preventive advice than those who declined. Second, we cannot rule out that the study setting influenced the secondary preventive care provided to the control group. The study team provided the GPs with LDL-C measurements that might not have been performed otherwise. Furthermore, it is possible that patients, by participating in the study, became more prone to contact their GP for secondary preventive assessment. The possible influence on the control group (simply by participating in a study) and the LDL-C measurements according to the study protocol may have led to an underestimation of the intervention effect. Third, regarding reasons for not reaching the target LDL-C value, documentation in the NAILED trial is less extensive for the control group than for the intervention group. The GPs who received test results from the NAILED follow-up had to sign them, but may not have documented any conclusions in the patients’ records.

## Conclusion

In this study, we have shown that during the first year after the introduction of a new cardiovascular secondary preventive guideline defining a lower LDL-C target value, fewer patients reached target regardless of nurse-based telephone follow-up or usual care. However, nurse-based telephone follow-up performed better than usual care, and the large proportion of LDL-C values above target in the intervention group was explained by inadequate treatment effect despite full-dose medication. This supports the hypothesis that nurse-based telephone follow-up according to the NAILED trial is an effective way to improve secondary preventive care after a cardiovascular event.

### Ethics approval and consent to participate

The study was approved by the Regional Ethics Committee, Umeå University (reference number Dnr-09-142 M). All eligible patients were informed about the study and those who agreed to participate were asked to give a written informed consent.

### Consent for publication

Not applicable.

### Availability of data and materials

Patient level data will be available on request, provided that an approval is given from the Regional Ethics Review Board at Umea University, Sweden.

## Abbreviations

ACS: acute coronary syndrome
BP: blood pressure
CVD: cardiovascular disease
DM: diabetes mellitus
GP: general practitioner
LDL-C: low-density lipoprotein cholesterol
NAILED: Nurse-Based Age-Independent Intervention to Limit Evolution of Disease
TIA: transient ischemic attack

## References

[1] Hackam DG, Spence JD (2007). Combining multiple approaches for the secondary prevention of vascular events after stroke: a quantitative modeling study. Stroke.

[2] Mancia G, Fagard R, Narkiewicz K, Redon J, Zanchetti A, Bohm M (2013). 2013 ESH/ESC guidelines for the management of arterial hypertension: the Task Force for the Management of Arterial Hypertension of the European Society of Hypertension (ESH) and of the European Society of Cardiology (ESC). Eur Heart J.

[3] Perk J, De Backer G, Gohlke H, Graham I, Reiner Z, Verschuren M (2012). European Guidelines on cardiovascular disease prevention in clinical practice (version 2012). The Fifth Joint Task Force of the European Society of Cardiology and Other Societies on Cardiovascular Disease Prevention in Clinical Practice (constituted by representatives of nine societies and by invited experts). Eur Heart J.

[4] Kernan WN, Ovbiagele B, Black HR, Bravata DM, Chimowitz MI, Ezekowitz MD (2014). Guidelines for the prevention of stroke in patients with stroke and transient ischemic attack: a guideline for healthcare professionals from the American Heart Association/American Stroke Association. Stroke.

[5] Smith SC, Benjamin EJ, Bonow RO, Braun LT, Creager MA, Franklin BA (2011). AHA/ACCF Secondary Prevention and Risk Reduction Therapy for Patients with Coronary and other Atherosclerotic Vascular Disease: 2011 update: a guideline from the American Heart Association and American College of Cardiology Foundation. Circulation.

[6] Dallongeville J, Banegas JR, Tubach F, Guallar E, Borghi C, De Backer G (2012). Survey of physicians’ practices in the control of cardiovascular risk factors: the EURIKA study. Eur J Prev Cardiol.

[7] Heidrich J, Behrens T, Raspe F, Keil U (2005). Knowledge and perception of guidelines and secondary prevention of coronary heart disease among general practitioners and internists. Results from a physician survey in Germany. Eur J Cardiovasc Prev Rehabil.

[8] Hobbs FD, Erhardt L (2002). Acceptance of guideline recommendations and perceived implementation of coronary heart disease prevention among primary care physicians in five European countries: the Reassessing European Attitudes about Cardiovascular Treatment (REACT) survey. Fam Pract.

[9] Alvarez-Sabin J, Quintana M, Hernandez-Presa MA, Alvarez C, Chaves J, Ribo M (2009). Therapeutic interventions and success in risk factor control for secondary prevention of stroke. J Stroke Cerebrovasc Dis.

[10] Bhatt DL, Steg PG, Ohman EM, Hirsch AT, Ikeda Y, Mas JL (2006). International prevalence, recognition, and treatment of cardiovascular risk factors in outpatients with atherothrombosis. JAMA.

[11] Saposnik G, Goodman SG, Leiter LA, Yan RT, Fitchett DH, Bayer NH (2009). Applying the evidence: do patients with stroke, coronary artery disease, or both achieve similar treatment goals?. Stroke.

[12] Kotseva K, Wood D, De Bacquer D, De Backer G, Ryden L, Jennings C (2015). EUROASPIRE IV: A European Society of Cardiology survey on the lifestyle, risk factor and therapeutic management of coronary patients from 24 European countries. Eur J Prev Cardiol.

[13] Buckley BS, Byrne MC, Smith SM (2010). Service organisation for the secondary prevention of ischaemic heart disease in primary care. Cochrane Database Syst Rev.

[14] Lager KE, Mistri AK, Khunti K, Haunton VJ, Sett AK, Wilson AD (2014). Interventions for improving modifiable risk factor control in the secondary prevention of stroke. Cochrane Database Syst Rev.

[15] McAlister FA, Majumdar SR, Padwal RS, Fradette M, Thompson A, Buck B (2014). Case management for blood pressure and lipid level control after minor stroke: PREVENTION randomized controlled trial. CMAJ.

[16] Neubeck L, Redfern J, Fernandez R, Briffa T, Bauman A, Freedman SB (2009). Telehealth interventions for the secondary prevention of coronary heart disease: a systematic review. Eur J Cardiovasc Prev Rehabil.

[17] Mooe T, Bjorklund F, Graipe A, Huber D, Jakobsson S, Kajermo U (2014). The Nurse-Based Age Independent Intervention to Limit Evolution of Disease After Acute Coronary Syndrome (NAILED ACS) Risk Factor Trial: Protocol for a Randomized Controlled Trial. JMIR Res Protoc.

[18] Mooe T, Bergstrom L, Irewall AL, Ogren J (2013). The NAILED stroke risk factor trial (nurse based age independent intervention to limit evolution of disease after stroke): study protocol for a randomized controlled trial. Trials.

[19] Shojania KG, Ranji SR, McDonald KM, Grimshaw JM, Sundaram V, Rushakoff RJ (2006). Effects of quality improvement strategies for type 2 diabetes on glycemic control: a meta-regression analysis. JAMA.

[20] Lear SA, Spinelli JJ, Linden W, Brozic A, Kiess M, Frohlich JJ (2006). The Extensive Lifestyle Management Intervention (ELMI) after cardiac rehabilitation: a 4-year randomized controlled trial. Am Heart J.

[21] Redfern J, Briffa T, Ellis E, Freedman SB (2009). Choice of secondary prevention improves risk factors after acute coronary syndrome: 1-year follow-up of the CHOICE (Choice of Health Options In prevention of Cardiovascular Events) randomised controlled trial. Heart.

[22] Vale MJ, Jelinek MV, Best JD, Dart AM, Grigg LE, Hare DL (2003). Coaching patients On Achieving Cardiovascular Health (COACH): a multicenter randomized trial in patients with coronary heart disease. Arch Intern Med.

[23] Robinson JG, Nedergaard BS, Rogers WJ, Fialkow J, Neutel JM, Ramstad D (2014). Effect of evolocumab or ezetimibe added to moderate- or high-intensity statin therapy on LDL-C lowering in patients with hypercholesterolemia: the LAPLACE-2 randomized clinical trial. JAMA.

[24] Cannon CP, Blazing MA, Giugliano RP, McCagg A, White JA, Theroux P (2015). Ezetimibe Added to Statin Therapy after Acute Coronary Syndromes. N Engl J Med.

[25] Graham IM, Stewart M, Hertog MG (2006). Cardiovascular Round Table Task F. Factors impeding the implementation of cardiovascular prevention guidelines: findings from a survey conducted by the European Society of Cardiology. Eur J Cardiovasc Prev Rehabil.

[26] Hibble A, Kanka D, Pencheon D, Pooles F (1998). Guidelines in general practice: the new Tower of Babel?. BMJ.

[27] Mampuya WM, Frid D, Rocco M, Huang J, Brennan DM, Hazen SL (2013). Treatment strategies in patients with statin intolerance: the Cleveland Clinic experience. Am Heart J.

[28] Zhang H, Plutzky J, Skentzos S, Morrison F, Mar P, Shubina M (2013). Discontinuation of statins in routine care settings: a cohort study. Ann Intern Med.

